# Multi-Target Risk Assessment of Potentially Toxic Elements in Farmland Soil Based on the Environment-Ecological-Health Effect

**DOI:** 10.3390/ijerph15061101

**Published:** 2018-05-28

**Authors:** Zhongyang Wang, Bo Meng, Wei Zhang, Jinheng Bai, Yingxin Ma, Mingda Liu

**Affiliations:** College of Land and Environmental Science, Shenyang Agricultural University, Shenyang 110866, China; wzysyau@163.com (Z.W.); Mbcash2015@163.com (B.M.); Zhangweilm04@163.com (W.Z.); bjhsyau@163.com (J.B.); 15704709951@163.com (Y.M.)

**Keywords:** China, ecological hazard, farmland, PMF model, risk assessment

## Abstract

There are potential impacts of Potentially Toxic Elements (PTEs) (e.g., Cd, Cr, Ni, Cu, As, Zn, Hg, and Pb) in soil from the perspective of the ecological environment and human health, and assessing the pollution and risk level of soil will play an important role in formulating policies for soil pollution control. Lingyuan, in the west of Liaoning Province, China, is a typical low-relief terrain of a hilly area. The object of study in this research is the topsoil of farmland in this area, of which 71 soil samples are collected. In this study, research methods, such as the Nemerow Index, Potential Ecological Hazard Index, Ecological Risk Quotient, Environmental Exposure Hazard Analysis, Positive Matrix Factorization Model, and Land Statistical Analysis, are used for systematical assessment of the pollution scale, pollution level, and source of PTEs, as well as the ecological environmental risks and health risks in the study area. The main conclusions are: The average contents of As, Cd, Cr, Cu, Hg, Zn, Ni, and Pb of the soil are 5.32 mg/kg, 0.31 mg/kg, 50.44 mg/kg, 47.05 mg/kg, 0.03 mg/kg, 79.36 mg/kg, 26.01 mg/kg, and 35.65 mg/kg, respectively. The contents of Cd, Cu, Zn, and Pb exceed the background value of local soil; Cd content of some study plots exceeds the National Soil Environmental Quality Standard Value (0.6 mg/kg), and the exceeding standard rate of study plots is 5.63%; the comprehensive potential ecological hazard assessment in the study area indicates that the PTEs are at a slight ecological risk; probabilistic hazard quotient assessment indicates that the influence of PTEs on species caused by Cu is at a slight level (*p* = 10.93%), and Zn, Pb, and Cd are at an acceptable level. For the ecological process, Zn is at a medium level (*p* = 25.78%), Cu is at a slight level (19.77%), and the influence of Cd and Pb are acceptable; human health hazard assessment states that the Non-carcinogenic comprehensive health hazard index HI = 0.16 < 1, indicating that PTEs in soil have no significant effect on people’s health through exposure; the PMF model (Positive Matrix Factorization) shows that the contribution rates of agricultural source, industrial source, atmospheric dust source, and natural source are 13.15%, 25.33%, 18.47%, and 43.05%, respectively.

## 1. Introduction

Since the “Minamata disease” and “Itai-itai disease” was found to be caused by Hg and Cd pollution in Japan in the 1950’s, Potentially Toxic Elements (PTEs) pollution has caused widespread concern around the world [1]. The accumulation of PTEs in soil is becoming more and more serious due to human activities such as industrial wastes, the unreasonable application of chemical fertilizers and pesticides, and landfill [2,3]. Adamcova [2] and Barbara [3] respectively assessed Potentially Toxic Elements pollution in landfill soil of the Czech Republic and Warsaw, and the results proved that PTEs (Cr, Cu, Pb, Cd, Zn) were of ecological toxicity and environmental harm. Accumulation in the soil, biological toxicity, and the irreversibility of heavy metal [4] will not only cause serious damage to the ecological security [2,3,5,6], leading to the decrease of the quality of the crops [7], but also give rise to long-term hazards [8] through soil, skin contact, respiratory inhalation, and other exposure approaches to human health (especially children) and even cause serious harm to human health through food chains [9,10,11].

The contamination of farmland soil in China has attracted much attention in recent years [12,13,14,15]. According to the report, about 10% arable land exceeds the heavy metal standard in China. Among this land, the exceeding standard proportion of Cd is higher than other elements, accounting for about 40% of the total exceeding plots [16]. China’s Ministry of Environmental Protection and the Ministry of Land and Resources released the bulletin of the “National Soil Pollution Survey” in 2014, pointing out that the soil environment in China was not optimistic, and the quality of the arable soil environment was worth serious consideration [17]. It demonstrates that 45% of the 29 provinces, autonomous regions, and municipalities in mainland China present moderate potential non-carcinogenic hazards to children, but they do not exceed the acceptable level [18]. According to the results of the spatial distribution, contamination, and ecological hazard assessment of the PTEs of farmland soils in the Karashahar–Baghrash oasis, northwest China, it shows that the average content of Cd exceeds the national standard of China by 10.80 times in this area and the moderately/seriously polluted areas with moderate potential ecological hazards distribute in the southern area [19]. For this reason, under the circumstance of the current soil environment, the protection of the environment and the protection of health will become the most urgent needs of the state and the people.

As is known, a cumulative process forms Potentially Toxic Elements (PTEs) pollution. Therefore, understanding the status of PTEs contamination and evaluating the sources of pollution and the possibility of contamination are the precondition for the prevention and control of PTEs contamination. Currently, there are many methods employed for the evaluation of PTEs pollution, such as the Nemerow’s pollution index [20], enrichment factors [21], the index of Geoaccumulation [22], and the potential ecological hazard index [23]; the statistical evaluation based on the geographic information system (GIS) [24]; source analysis based on the receptor model and human health hazard assessment [25,26,27]; and other comprehensive methods. However, there is no unified standard of different evaluation methods, and every method has certain advantages and disadvantages. In order to assess the pollution of PTEs (e.g., Cd, Cr, Ni, Cu, As, Zn, Hg, and Pb) scientifically and reasonably, environmental-ecological-health should be regarded as a whole, and then take into account the regional environmental hazard, regional ecological hazard, and local residents hazard caused by PTEs. Therefore, the Hakanson potential ecological index method, the ecological hazard quotient method recommended by the U.S. Environmental Protection Agency (U.S. EPA), the health hazard model, and the PMF model are applied to explore the environmental hazard level and main sources of PTEs in the study area. We aim to integrate a set of evaluation methods and measures to combine environment- ecological-health.

The purpose of this study is to find out the current situation of Potentially Toxic Elements (PTEs) in farmland soil in the Lingyuan area, assess the harm to the local ecological environment and the hazards to residents’ health caused by PTEs pollution, and explore the sources of PTEs pollution.

## 2. Materials and Methods

### 2.1. Study Area General Situation

Lingyuan, in the west of Liaoning Province, is a typical low-relief terrain of a hilly area, where ravines and gullies criss-cross. The terrain is high in the northwest and low in the southeast. Geotectonically, it is part of the North China platform, most of which belongs to Yanshan fold belts. Weathered rock residues (mainly composed of Granite and Basalt) are the main component of local parent material, which account for about 57% of the total parental material. The soil formed by these parent materials is relatively thin and non-cultivated. The soil types are mainly brown soil, cinnamon soil, and meadow soil. The average annual temperature is 10 °C, and the annual precipitation is 450~580 mm. It belongs to the continental monsoon climate in the north temperate zone, and the interannual temperature difference is large, with a hot rainy season, a large temperature difference between day and night, and less rainfall. There are abundant mineral resources in the region, and more than 50 kinds of metal and non-metallic mineral reserves have been explored. Because of the inconsistencies in the distribution of metal mining areas in the region, there are obvious differences in the impact of the soil environment in different villages and towns.

### 2.2. Sample Collection and Data Source

**Figure 1:**
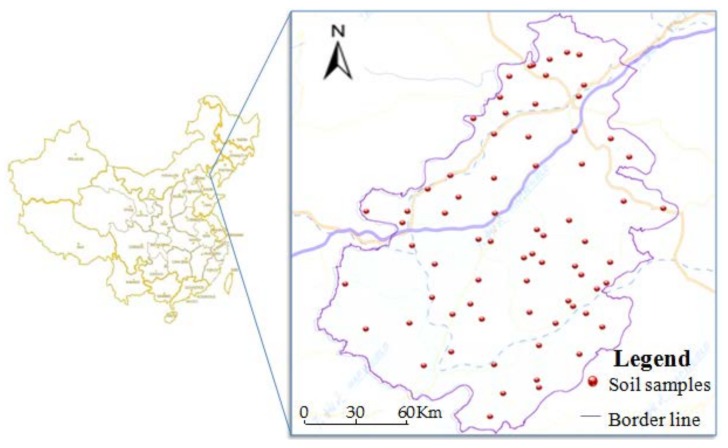
Distribution of soil sampling sites in the study area.

Sample collection was completed between July and September of 2016. According to the regulation of Soil Environmental Monitoring Technology (HJ/T 166-2004) [28], we used the uniform point method to collect the plowed soil of 0–20 cm, and then located the points in ArcGIS10.2 (Environmental Systems Research Institute, Inc, USA) and LocaSpaceViewer software (Beijing 3D vision technology Co., Ltd, China) and located accurately with GPS in sampling. Based on the previous research and collection of historical data, 71 samples were collected in this study (see Figure 1), and the targeted detection items were Cd, Cu, Zn, Pb, As, Hg, Ni, and Cr. According to the test method in the GB 15618 trial, the soil sample was dried in a ventilated and cool place and passed over 100 sieves. Each sample had a weight of 0.1–0.2 g with 1/10,000 balance scales (accurately record data) in a polytetrafluoroethylene digestion tank. Soil samples were digested by the HCl-HNO_3_-HClO_4_-HF method. Then, three parallels were set for each sample. At the same time, the quality control of the whole blank sample and the national standard reference material (GSS-3) was analyzed. The results of the samples were all within the allowable error range. The reagents used in the analysis were all analytically pure. Soil samples were digested with a Microwave digestion instrument (CEM Inc., Matthews, NC, USA) and prepared for the determination of elements. The soil samples Hg and As were measured by HG-AES, while Cr, Cu, Ni, Pb, Zn, and Cd were measured by Inductively Coupled Plasma Mass Spectroscopy (ICP-MS, PerkinElmer, Waltham, MA, USA). The Instrument Detection Limit (mg/kg) of Cu, Zn, Pb, Cd, Ni, Cr, Hg, and As is: 0.04, 0.04, 0.1, 0.001, 0.04, 0.04, 0.001, and 0.01, respectively.

### 2.3. Ecological Hazard Assessments of Potentially Toxic Elements in Soil 

#### 2.3.1. Potential Ecological Hazard Index Method

The expression of the single element ecological hazard index *E_r_^i^* and the comprehensive potential ecological hazard index (RI) of a variety of Potentially Toxic Elements is shown as:

(1) Eri=Tri × Cri=Tri × CCni

(2) $$RI=\sum_{i}^{n}E_{r}^{i}$$

$T_{r}^{i}$ is the toxicity response parameter of the biological toxicity response factors of different metals, stating the toxicity level and the biological sensitivity to its pollution. *C* is the measured value and $C_{n}^{i}$ is the reference value. $E_{r}^{i}$ is a single potential ecological hazard index and RI represents the potential ecological hazard factor of a sample of PTEs.

Based on the Hakanson grading standard, we should consider the toxicity coefficient of eight pollutants and the calculation should depend on the number of pollutants and the number of PTEs with the maximum toxicity coefficient of the eight elements [29]. This study was only aimed at four kinds of PTEs, so we adjusted the evaluation criteria according to the number and types of PTEs to ensure that the evaluation results were more accurate. The adjustment method of potential ecological hazard assessment standard: define the unit toxicity coefficient grading value RI = 150 (Hakanson first grade classification limit value)/133 (the total toxicity coefficient of eight pollutants) = 1.13 [30]. The largest PTEs toxic coefficient in this paper is Cd (30), the total toxicity coefficient of the four kinds of PTEs is 41, and the adjusted first grade classification limit value is RI = 41 × 1.13 ≈ 46. The other grade classification limit values are twice that of the value of the upper level. Table 1 demonstrates the comparison of the $E_{r}^{i}$ and RI values before and after the adjustment.

#### 2.3.2. Ecological Hazard Quotient Evaluation Method

The ecological hazard can be expressed as the probability of the environmental concentration (EC) exceeding the bio-sensitive concentration (SS) [31]. The exceeding probability may be considered as the negative effect probability of pollutants to soil ecological species or the soil ecological process, that is, the probability hazard of EC/SS > 1 can be expressed as:

(3) P(EC≥SS)=P(RQ≥1)

(4) Risk=P[lg(EC>SS)>0]=P[lg(EC)−lg(SS)>0]

### 2.4. Health Hazard Assessments of Potentially Toxic Elements in Soil

#### 2.4.1. Evaluation Model

The measurement of non-carcinogenic daily average exposure in three exposing ways is as follows:

(5) $$ADD_{ing}=\frac{c\;\times \;IngR\;\times \;CF\;\times \;EF\;\times \;ED}{BW\;\times \;AT}$$

(6) $$ADD_{inh}=\frac{c\;\times \;InhR\;\times \;EF\;\times \;ED\;\times \;CF\;\times \;FSPO\;\times \;PLAF\;\times \;PM10}{BW\;\times \;AT}$$

(7) $$ADD_{derm}=\frac{c\;\times \;SA\;\times \;CF\;\times \;SL\;\times \;ABS\;\times \;EF\;\times \;ED}{BW\;\times \;AT}$$

*ADD_ing_*, *ADD_inh_*, and *ADD_derm_* are daily average exposure measurements of soil uptake, respiratory inhalation, and skin absorption (mg/kg·d), respectively. The parameters are summarized in Table 2. Considering the particularity of children, this paper selects children as the hazard assessment objective [32]. On one hand, children’s weight is less than that of adults; on the other hand, children are more exposed to soil because of more outdoor activities, which makes them have a greater health hazard than adults.

#### 2.4.2. Hazard Characterization

According to the results of different exposures, the non-carcinogenic health hazard of PTEs in soil is calculated as follows:

(8) $$HQ_{i}=\overset{3}{\underset{j=1}{\sum }}\frac{ADD_{ij}}{RfD_{ij}}\;\;\;$$

(9) $$HI=\overset{4}{\underset{i=1}{\sum }}HQ_{i}$$

In the formula, *HQ* is the single health hazard index of non-carcinogenic element *i*; *ADD_ij_* refers to the daily exposure result of non-carcinogenic element *i* through the exposure way *j*, mg/(kg·d); and *RfD_ij_* represents the daily average exposure reference dose of non-carcinogenic element *i* through the exposure way *j*, which is considered by US EPA as having no predictable harmful effects on human beings after lifetime exposure (see Table 3) [33]. HI is the total non-carcinogenic hazard index of four PTEs through three exposing ways. When *HI* or *HQ_i_* > 1, the PTEs are a non-carcinogenic hazard to human health; when both *HQ_i_* and *HI* are less than 1, the hazard to non-carcinogenic health can be ignored.

### 2.5. PMF Receptor Model

The PMF receptor model has been widely used by scholars all around the world. The basic equation is as follows:

(10) $$E_{ij}=X_{ij}-Y_{ij}=X_{ij}-\sum_{h=1}^{P}G_{ih}F_{hj}\left(i=1,\ldots ,j=1,\ldots ,n\right)$$

In the equation, *E_ij_* is the concentration of element *j* in the sample *i*; *G_ij_* is the contribution of sample *i* to the source *h*, that is, the source sharing rate matrix; and *F_hj_* is the concentration of the pollutant *j* in the source *h*, that is, the source component spectrum matrix. The objective function *Q* is:

(11) $$Q=\overset{m}{\underset{i=1}{\sum }}\overset{n}{\underset{j=1}{\sum }}\left(\frac{E_{ij}}{\sigma_{ij}}\right)$$

In the equation, σ*_i__j_* represents *E_i__j_* uncertainty. When the concentration of PTEs is less than or equal to the corresponding method detection limit (MDL), the formula of uncertainty is calculated as: Unc=5/6 × MDL . When the concentration of PTEs is greater than the corresponding MDL, the value of the uncertainty is: $\;\mathrm{Unc}={\left[{\left(\sigma \;\times \;c\right)}^{2}+{\left(\mathrm{MDL}\right)}^{2}\right]}^{1/2}$. 

### 2.6. Statistical Methods

The sample distribution map of the research area is completed by ArcGIS10.2 and LocaSpaceViewer, the statistics and analysis of the data are conducted through SPSS18.0 (SPSS Inc., IBM, Chicago, IL, USA), the source analysis of PTEs in soil is completed by PMF5.0 (Sonoma Technology Inc., CA, USA), and the other data processing is completed by Excel 2013 (Microsoft, Redmond, WA, USA).

## 3. Results and Discussion

### 3.1. Descriptive Statistics of Potentially Toxic Elements Content in Soil

According to the statistical analysis, it is found that the pH value of the soil in the study area is mainly between 7–8.3, and it is attributed as alkaline soil. Comparing the content of PTEs in soil with the local soil background value [34], the content of Cd shows the most significant difference. The mean value of Cd is 4.4 times that of the background value, followed by Cu and Pb, of which the mean contents are 2.1 and 1.7 times higher than that of the background values. The ratios of Cd, Pb, Cu, Zn, Ni, Hg, and Cr in soil samples exceeding the background value are 92.96%, 63.3%, 84.51%, 47.89%, 33.80%, 40.85%, and 19.72%, respectively.

**Figure 2:**
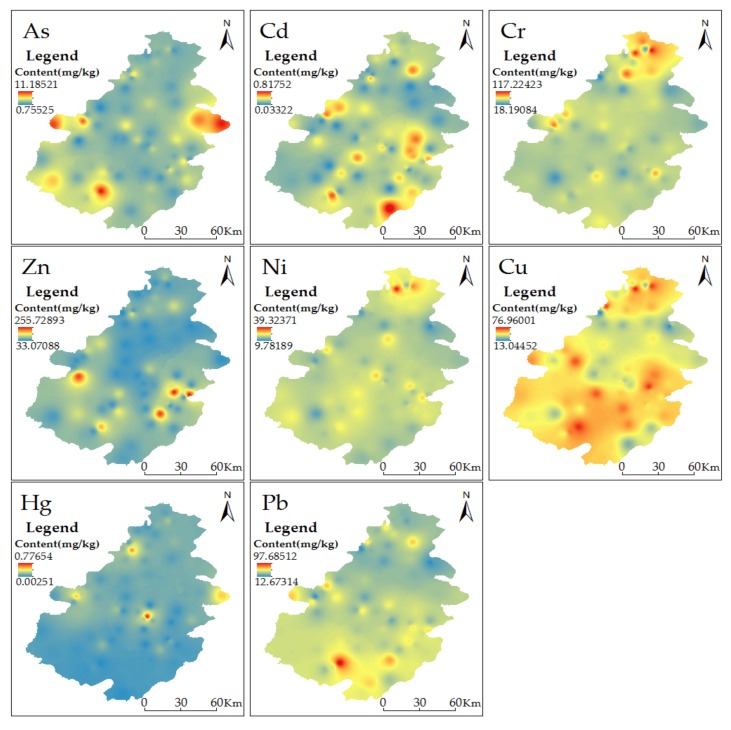
Spatial distribution of Potentially Toxic Elements concentrations.

It demonstrates that the soil in the study area displays the accumulation of PTEs in varying degrees. When there are outliers or very different values, Inverse Distance Weighting (IDW) interpolation gives better results than the model kriging [35,36]. Therefore, this study uses the Land statistical analysis module (IDW) of ArcGIS to interpolate the content of each element, which can better illustrate the content and distribution of PTEs in the study area. We can see the spatial distribution of PTEs in soil from Figure 2. The distribution of Ni and Cr has some spatial similarity: the high value area of both appears in the northern part of the region. Because the average content of Ni and Cr is lower than the soil background value in the study area, it is concluded that this distribution may be related to the soil parent material of the region. The high value area of Hg appears in the northwest part of the region and the content variability is prominent. Because the main source of Hg is atmospheric sedimentation, it is deduced that the residents’ consummation of coal may be a major reason for the formation of the high value area of Hg. The high value area of As appears in the west and east. Since the distribution is relatively dispersed, the content value is generally low, and there is no pollution phenomenon, it is concluded that As distribution may be related to the soil parent material. The content of Cd, Cu, Zn, and Pb is high in the southeast, which is basically consistent with the distribution of minerals in the study area. Moreover, the industrial types in the area are complex and diverse, and the traffic network is well-developed. Research has shown that factory production activities [37], vehicle exhaust [38], and tire wear [39] will cause PTEs (e.g., Cd, Cu, Zn, and Pb) to accumulate in the soil. Therefore, it is concluded that the main reason for the high value area of Cd, Cu, Zn, and Pb is the imperfect mining technology, industrial emission, and transportation.

According to the “Soil Environmental Quality Standard” (GB 15618) Trial Edition (2017), the rate of Cd exceeds the standard by 5.63%, and the maximum values of other elements are less than the national standard. The cumulative pollution index method [40] was used to analyze the accumulation of PTEs in soil, as shown in Table 4. According to the grading of the Muller index for PTEs pollution, it was concluded that Cd constitutes moderate pollution in the study area, Cu constitutes slight pollution, and the rest of the elements are not polluted, which indicates that Cd and Cu are more seriously accumulated in soil. Combining the local soil background value, the national standard value, and the evaluation results of the cumulative pollution index method, it can be concluded that there exists a potential hazard of PTEs Cd, Zn, Cu, and Pb in the study area. Therefore, the hazard assessment of these four elements has been carried out in this paper.

### 3.2. Assessment of Soil Ecological Hazard in the Study Area

#### 3.2.1. Potential Ecological Hazard Assessment

Table 5 is the potential ecological hazard assessment of PTEs in the study area. The PTEs in the study area are in a slight ecological hazard state, and the potential ecological hazard is declining in the following order: Cd > Cu > Pb > Zn. Among them, one of the Cd samples is a moderate ecological hazard, and the hazard values of the other elements exhibit a slight ecological hazard level. In the whole study area, all the indicators of all the samples have a potential ecological hazard index ranging between 5.2–44.95, which indicates a slight ecological hazard.

#### 3.2.2. Ecological Hazard Quotient Evaluation

The data of the PTEs content after removing the peak value is all in line with a log-normal distribution, and the probability density function can be expressed by the probability density function of normal distribution:

(12) $$F\left(RCds\right)=0.73exp\left[\frac{-{\left(lg\left(EC_{Cd}/Cds\right)+1.72\;\right)}^{2}}{0.60}\right]$$

(13) $$F\left(RCdp\right)=0.57exp\left[\frac{-{\left(lg\left(EC_{Cd}/Cdp\right)+2.67\right)}^{2}}{0.98}\right]$$

(14) $$F\left(RCus\right)=0.58exp\left[\frac{-{\left(lg\left(EC_{Cu}/Cus\right)+0.85\right)}^{2}}{0.94}\right]$$

(15) $$F\left(RCup\right)=0.60exp\left[\frac{-{\left(lg\left(EC_{Cu}/Cup\right)+0.56\right)}^{2}}{0.88}\right]$$

(16) $$F\left(RZns\right)=1.18exp\left[\frac{-{\left(lg\left(EC_{Zn}/Zns\right)+0.73\right)}^{2}}{0.23}\right]$$

(17) $$F\left(RZnp\right)=0.70exp\left[\frac{-{\left(lg\left(EC_{Zn}/Znp\right)+0.37\right)}^{2}}{0.65}\right]$$

(18) $$F\left(RPbs\right)=0.76exp\left[\frac{-{\left(lg\left(EC_{Pb}/Pbs\right)+1.16\right)}^{2}}{0.55}\right]$$

(19) $$F\left(RPbp\right)=0.67exp\left[\frac{-{\left(lg\left(EC_{Pb}/Pbp\right)+1.28\right)}^{2}}{0.71}\right]$$

In the formulas, *RCds*, *RCdp*, *RCus*, *RCup*, *RZns*, *RZnp*, *RPbs*, and *RPbp* represent the ecological hazard quotients of soil Cd, Cu, Zn, and Pb to biological species (*s*) and ecological processes (*p*), respectively. *EC_Cd_*, *EC_Cu_*, *EC_Zn_*, and *EC_Pb_* represent the content of Cd, Cu, Zn, and Pb in soil. *Cds*, *Cdp*, *Cus*, *Cup*, *Zns*, *Znp*, *Pbs*, and *Pbp* represent the invalid concentrations of Cd, Cu, Zn, and Cd for biological species (*s*) and ecological processes (*p*), respectively.

**Figure 3:**
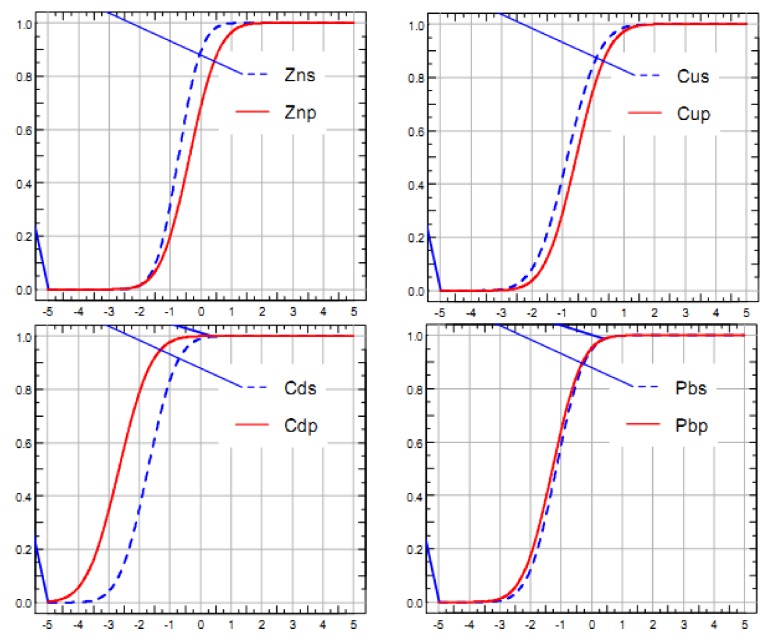
The curve of accumulation probability of the element hazard quotient.

Figure 3 is the curve of accumulation probability of the element hazard quotient (Cd, Cu, Zn, Pb). The *x*-coordinate is the range of values for the variable, and the ordinate is the cumulative probability. The analysis shows that the hazard ratio of impact on the species of Cd, Cu, Zn, and Pb is 0.87%, 10.93%, 1.58, and 1.29%; and the hazard ratio of impact on the ecological process of Cd, Cu, Zn, and Pb is 0.07%, 19.77%, 25.78%, 0.34%, respectively. It can be seen that Cu displays the highest hazard to species, followed by Zn, Pb, and Cd; and Zn exhibits the highest hazard to the ecological process, followed by Cu, Pb, and Cd. Generally speaking, the ecological hazard probability (*p*) is divided into five levels, namely, an acceptable level (*p* < 10%), slight impact (10% < *p* < 25%), moderate impact (25% < *p* < 50%), serious harm (50% < *p* < 75%), and extremely serious harm (75% < *p* < 100%). From the species point of view, Cu generates a slight impact, and Cd, Zn, and Pb are all at an acceptable level; from the ecological process point of view, Zn has a moderate impact, Cu exhibits a slight impact, and the effects of Cd and Pb are acceptable.

### 3.3. Health Risk Assessment and Source Analysis of Potentially Ttoxic Elements in the Study Soil

#### 3.3.1. Health Risk Assessment

Based on the average of PTEs content in farmland soil, the health risk assessment models and parameters are applied to calculate the average human health risk when considering different exposure ways. The results are shown in Table 6.

The exposure risk index of non-carcinogenic PTEs for children shows that the non-carcinogenic risk of each element is in the order of Pb > Cu > Zn > Cd. Exposure to PTEs in soil uptake is far higher than for the other two exposure ways, which can be understood by the fact that this is related to the daily activities of children [41]. The single hazard index HQ of Cd, Pb, Zn, and Cu for children in three exposure ways and the comprehensive health risk index HI are all less than 1, which indicates that the effect of the four non-carcinogenic PTEs on children’s health is not significant. Because children’s body weight is lighter than that of adults, the frequency of exposure to PTEs is higher than that of adults, and children have a higher hazard of non-carcinogenic PTEs exposure than adults under the same circumstance. Therefore, the hazard of adult exposure is also acceptable.

#### 3.3.2. Source analysis of Potentially Toxic Elements in Soil

**Figure 4:**
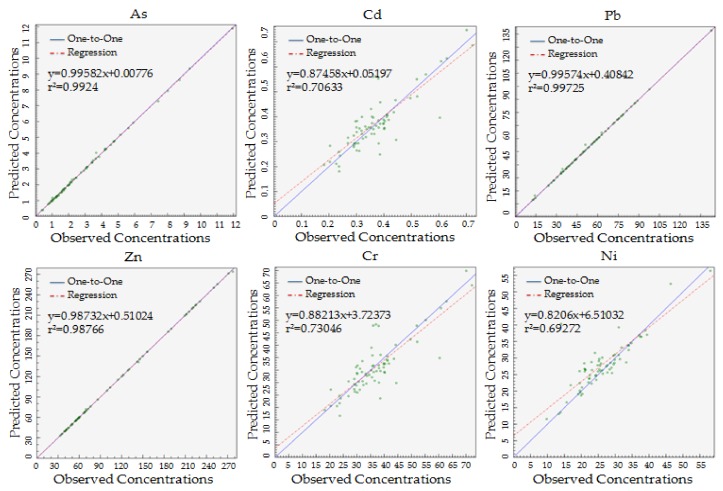
Fitting between elements observed concentrations and predicted concentrations by PMF.

By using the PMF model for source analysis, we need to input the element content and the element content uncertainty to reflect the Signal-to-Noise ratio (S/N) of the element. In this study, the S/N of Hg is small, defined as “Bad”, which should be eliminated. The value of Q is minimized and close to the theoretical value after multiple operations in order to ensure the correlation between the simulated results and the measured results. As shown in Figure 4, the r^2^ of each element is more than 0.6, so the correlation is good, which shows that the overall fitting effect of the PMF model is good and it meets the needs of the research.

The four best factors are analyzed by the PMF model. The contribution rate of each factor and the PTEs source component spectrum are shown in Table 7.

Factor 1 has a high contribution to the elements Cu, Zn, As, and Pb, while the large variation coefficient of Zn, Pb, and As shows that the human influence is relatively serious. In the process of sampling and investigation, it was found that pesticides, phosphate fertilizer, and manure are mainly used in agricultural cultivation. According to the literature, the irrational use of pesticides and fertilizers will lead to the accumulation of As in the soil, and the content of Cu, Zn, and Pb in manure is far higher than in other kinds of fertilizer. Related studies have also shown that the accumulation of Cu and Zn has a great relationship with the input of farming materials. Therefore, factor 1 can be interpreted as the source of input for agricultural activity.

The order of the contribution rate in factor 2 is Cd > Zn > Pb > Cu > As > Cr, where Cd has the highest contribution rate. Studies have shown that Cd is mainly derived from industrial waste emissions. From the spatial distribution of Cd in the study area, it can be seen that the accumulation of Cd is the largest and has a relative similarity with the distribution of local mineral resources. In the actual survey, it is found that there are some industrial and mining enterprises that will cause Cd input in the environment. Correlation analysis shows that Cd had a good correlation with Zn, and the study in this area shows that Zn content distribution had a significant positive correlation with mineral exploitation in this area. Therefore, factor 2 can be considered as an industrial source.

Factor 3 has a high contribution to Cu, Pb, and Zn. From the distribution of Zn, we can see that Zn is relatively high in the densely populated area of villages and towns, and the perennial wind direction in the study area is to the northwest or to south, which is also well fitted with Zn content. Pb is the main symbol of transportation. Vehicle exhaust in the sampling farmland will produce a certain amount of Pb, which falls to the soil surface with the dust. The smelting of plants around the farmland will produce heavy metal dust as well, and Pb will enter the surface of the soil along with the dry and wet deposition of the smelting dust. According to the relevant literature, the accumulation of Zn in the surface soil has a great relationship with transportation. The abrasion of auto tyres produces Zn, which falls into the surface soil along with the dust. Therefore, factor 3 can be interpreted as the atmospheric dust and the integrated source of transportation.

Factor 4 has the highest contribution to Cr, reaching 69.49%. The variation coefficient of soil Cr content in the study area is 28.14%, which indicates that the spatial variation of Cr content in this area is small, and the Cr in the soil is less influenced by human factors. Additionally, the average value of Cr is less than the background value of the study area, so it is concluded that the source of Cr is influenced by the natural soil parent material. Some analysis shows that Cr comes from the rock and enters into the soil and the soil parent material through weathering. Therefore, it is concluded that factor 4 should be the natural background.

The contribution rate of the agricultural source is 13.15%, the contribution rate of the industrial source is 25.33%, the contribution rate of the integrated source of transportation atmosphere sedimentation is 18.47%, and the contribution rate of natural source is 43.05%.

## 4. Conclusions

This research systematically studies the current situation of Potentially Toxic Elements and the sources in the farmland of the Lingyuan area. The average contents of Cd, Cu, Zn, and Pb in the study area are higher than the local background values, in which the Cd content exceeds the national standard by 5.63%. The single factor potential ecological hazards of Cd, Cu, Zn, and Pb are all at a slight hazard or below. The influence of PTEs on species caused by Cu is at a slight level, and Zn, Pb, and Cd are at an acceptable level. For the ecological process, Zn is at a medium level, Cu is at a slight level, and the influence of Cd and Pb is acceptable. Human health hazard assessment states that PTEs in soil have no significant effect on people’s health through exposure. The PMF model shows that the contribution rate of each source is ranked as follows: soil parent material sources > industrial sources > transportation and atmospheric sedimentation sources > agricultural sources.

However, to more precisely delineate the polluted areas, more attention needs to be paid to the following issues:

1. This study only evaluates the total content of PTEs in the soil environment, and an assessment of the available content of PTEs also needs to be considered.
2. In this study, the spatial scale of PTEs pollution analysis is large, and it can form the basis for multi-scale and multi-dimension research in further studies.

## Figures and Tables

**Table 1:** The classification of potential ecology hazard.

Parameter	Ecology Hazard	Slight	Intermediate	Strongly	Strongly–Extremely	Extremely
Hakanson	$E_{r}^{i}$	<40	40–80	80–160	160–320	≥320
	RI	<150	150–300	300–600	≥600	-
This research	$E_{r}^{i}$	<40	40–80	80–160	160–320	≥320
	RI	<46	46–92	92–184	≥184	-

**Table 2:** Health hazard assessment parameters of Potentially Toxic Elements.

Factor	Meaning	Value	Factor	Meaning	Value
IngR	Uptake of soil rate, mg/d	200	BW	Children’s weight, kg	16
InhR	Respiratory intake, m^3^/d	7.5	AT	Mean time of action, d	365 × 10
ABS	Skin absorption factor	0.001	FSPO	The proportion of soil particles in the air	0.5
EF	Exposed frequency, d/a	365	PLAF	Retention ratio	0.75
ED	Exposed year, a	10	PM10	Inhalable content, mg/m^3^	0.3
SA	Expose skin surface area, cm^2^	1600	SL	Adhesiveness of skin, mg·cm^−2^/d	0.2
c	Concentration, mg/kg	-	CF	Conversion fraction	1 × 10^−6^

**Table 3:** Health hazard assessment parameters of Potentially Toxic Elements.

Element		RfD (mg/kg d)	
	Soil Uptake	Respiratory Inhalation	Skin Absorption
Cd	0.1 × 10^−^^2^	0.1 × 10^−^^2^	0.1 × 10^−^^2^
Cu	0.038	0.012	0.038
Zn	0.3	0.3	0.3
Pb	3.5 × 10^−^^3^	0.525 × 10^−^^3^	0.352 × 10^−^^3^

**Table 4:** Summary statistics of Potentially Toxic Elements in soil (mg/kg) (*n* = 71).

Element	Range	Mean	Standard ^1^	Background	Muller Index	Formula
As	0.76–11.19	5.32	30	12.87	−2.19	$I_{\mathrm{geo}}=\log_{2}^{\left[Ci/(k\times Bi)\right]}$ Inside; *C_i_* means Elemental concentration (mg/kg); Bi means Background value (mg/kg); K = 1.5.
Cd	0.03–0.82	0.31	0.6	0.07	1.27	
Cr	18.1–117.2	50.44	250	64.33	−1.04	
Cu	13.4–76.9	47.05	100	22.33	0.34	
Hg	0.003–0.77	0.025	3.4	0.02	−0.71	
Zn	33.1–255.7	79.36	300	64.01	−0.42	
Ni	9.78–39.32	26.01	190	28.68	−0.78	
Pb	12.67–97.69	35.65	170	20.98	−0.05	
pH	7.02–8.73	8.06	-	-	-	

^1^ From: Ministry of Environmental Protection of the People’s Republic of China-(2017)1385.

**Table 5:** The classification of potential ecology hazard by Hakanson.

Parameter	Element	Toxic Parameters	Standard Value		$E_{r}^{i}$	
				Hazard Index	Average	Ranking
$E_{r}^{i}$	Cd	30	0.6	1.66–40.88	15.75	Slight
	Cu	5	100	0.65–3.85	2.35	
	Zn	1	300	0.11–0.88	0.27	
	Pb	5	170	0.32–2.87	1.07	
*RI*				5.2–44.95	19.44	Slight

**Table 6:** Assessment of health exposure risk.

Parameter	Soil Uptake	Respiratory Inhalation	Skin Absorption	Total Risk HQ
Cd	3.86 × 10^−3^	1.63 × 10^−5^	6.17 × 10^−6^	3.88 × 10^−3^
Cu	1.55 × 10^−2^	2.07 × 10^−4^	2.48 × 10^−5^	1.57 × 10^−2^
Zn	3.31 × 10^−3^	1.39 × 10^−5^	5.29 × 10^−6^	3.33 × 10^−3^
Pb	1.27 × 10^−1^	3.58 × 10^−3^	2.02 × 10^−3^	1.32 × 10^−1^
HI				1.56 × 10^−1^

**Table 7:** Source contribution for different elements by PMF.

Element	Source Profile (mg/kg)				Source Contribution (%)			
	Factor 1	Factor 2	Factor 3	Factor 4	Factor 1	Factor 2	Factor 3	Factor 4
As	1.29	0.21	0.86	3.57	15.43	8.11	9.2	67.26
Cd	0.04	0.13	0.001	0.09	15.72	45.19	7.24	31.85
Cr	3.95	8.13	11.1	39.7	4.47	7.33	14.71	73.49
Cu	9.91	10.7	7.49	19.32	21.48	27.5	16.9	34.12
Pb	4.43	9.33	6.45	7.61	18.14	33.82	21.63	26.41
Zn	7.25	21.83	13.47	18.23	12.47	36.71	23.9	26.92
Ni	1.26	3.54	7.87	13.33	4.32	18.68	35.73	41.27
